# Genetic Characteristics of Japanese Clinical *Listeria monocytogenes* Isolates

**DOI:** 10.1371/journal.pone.0122902

**Published:** 2015-03-31

**Authors:** Satoko Miya, Hajime Takahashi, Miku Nakagawa, Takashi Kuda, Shizunobu Igimi, Bon Kimura

**Affiliations:** 1 Department of Food Science and Technology, Faculty of Marine Science, Tokyo University of Marine Science and Technology, Tokyo, Japan; 2 Division of Biomedical Food Research, National Institute of Health Sciences, Tokyo, Japan; Institut National de la Recherche Agronomique, FRANCE

## Abstract

*Listeria monocytogenes* causes foodborne illnesses through consumption of ready-to-eat foods. Although 135-201annual listeriosis cases have been estimated in Japan, the details regarding the clinical isolates such as infection source, virulence level, and other genetic characteristics, are not known. In order to uncover the trends of listeriosis in Japan and use the knowledge for prevention measures to be taken, the genetic characteristics of the past human clinical isolates needs to be elucidated. For this purpose, multilocus tandem-repeat sequence analysis (MLTSA) and multi-virulence-locus sequence typing (MVLST) were used in this study. The clinical isolates showed a variety of genetically distant genotypes, indicating they were from sporadic cases. However, the MVLST profiles of 7 clinical isolates were identical to those of epidemic clone (EC) I isolates, which have caused several serious outbreaks in other countries, suggesting the possibility that they have strong virulence potential and originated from a single outbreak. Moreover, 6 Japanese food isolates shared their genotypes with ECI isolates, indicating that there may be risks for listeriosis outbreak in Japan. This is the first investigational study on genetic characteristics of Japanese listeriosis isolates. The listeriosis cases happened in the past are presumably sporadic, but it is still possible that some isolates with strong virulence potential have caused listeriosis outbreaks, and future listeriosis risks also exist.

## Introduction

*Listeria monocytogenes* is the cause of an important disease worldwide, mostly resulting from consumption of contaminated ready-to-eat (RTE) food. In Japan, no official statistics exist on the incidence rates of listeriosis because of the lack of a mandatory notification system. Consequently, only one foodborne listeriosis outbreak has been officially reported to date [1]. On the other hand, Japan Nosocomial Infections Surveillance (JANIS) estimated that 135–201 listeriosis cases occur every year in Japan (2008–2011), which is equivalent to 1.40 cases in a million people [2]. Compared to the previous estimation of 0.65 cases in a million people every year during 1997–2002 [3], the infection rate is increasing currently. However, the isolates from these clinical cases have never been investigated. Thus, the infection source, virulence level, and other characteristics of the isolates are not known.

The virulence potential and ecology of these isolates can be predicted to a large extent by using typing methods. For example, serotype 4b isolates have been responsible for most human listeriosis epidemics and a majority of human sporadic cases [4,5], whereas serotype 1/2a strains are mostly isolated from food related sources [6]. In addition, among the 4 evolutionary lineages [7–11], lineage I strains are significantly over-represented among human listeriosis isolates [12–14], while lineage II strains are more common in foods and food-processing environments. The correlation between the strain subtype and the virulence and ecological differences indicates that genotyping methods would be meaningful in clarifying the virulence characteristics of strains [15,16].

In this study, we performed molecular typing to genetically characterize Japanese clinical *L*. *monocytogenes* isolates. Two sequence-based typing methods, multilocus tandem-repeat sequence analysis (MLTSA) [17] and multi-virulence-locus sequence typing (MVLST) [18], were used to evaluate any possible distinctive trend among the isolates from the Japanese listeriosis cases.

## Materials and Methods

### *L*. *monocytogenes* isolates used

A total of 158 *L*. *monocytogenes* isolates were tested in this study, including 95 food and 21 listeriosis case isolates from Japan, and 42 clinical isolates from other countries, mainly the United States (S1 Table). The Japanese food isolates comprised of 61 RTE seafood isolates [19–21], 32 meat isolates (including 10 isolates from imported meat) [20,22], and 2 cutting board isolates. The Japanese clinical isolates had been collected by the Japanese National Institute of Health Sciences (NIHS isolates). Ethical approval was not required as the clinical isolates were collected as part of standard patient care. Among the clinical isolates from other countries, the FSL isolates had been kindly provided by Dr. Martin Wiedmann (Cornell University, Ithaca NY) and the others had been purchased from the American Type Culture Collection (ATCC; Manassas, Va), Collection de l’Institut Pasteur (CIP: Paris, France), and National Collection of Type Cultures (NCTC; London, United Kingdom). Serotypes of the isolates were analyzed by the conventional slide agglutination method, but serotype information for the NIHS and FSL isolates was provided by the Japanese NIHS and Cornell University, respectively.

### Lineage designation

*L*. *monocytogenes* are known to be grouped into 4 distinct phylogenetic lineages. Each of the 158 isolates used in this study was categorized into the lineages using a previously described method [23] involving multiplex PCR to produce a lineage-specific sized band on electrophoresis gels.

### MVLST

The MVLST method used in this study was developed by Zhang et al. [18]. Briefly, 6 *L*. *monocytogenes* virulence and virulence-associated genes (*prfA*, *inlB*, *inlC*, *dal*, *lisR*, and *clpP*) were PCR-amplified and sequenced using the ABI PRISM 3100 Genetic Analyzer (Applied Biosystems, Foster City, CA, USA). For each locus, DNA sequences that differed by at least one nucleotide were assigned different arbitrary allele numbers [24] that were entered into the BioNumerics v.4.0 software (Applied Maths, Sint-Martens-Latem, Belgium) for phylogenetic analysis.

### MLTSA

Previously we developed a typing method for *L*. *monocytogenes* by using 3 tandem-repeat regions (TR1, TR2, and TR3) [17] that could be used to discriminate strains on the basis of nucleotide sequence differences in these regions. We followed this method in the present study. The target regions were PCR-amplified and sequenced, and allele numbers were assigned to each isolate as in the abovementioned MVLST method. The data obtained were analyzed using the BioNumerics software.

## Results and Discussion

### Discriminatory ability of MLTSA—MVLST combination method

In this study, all the 158 isolates analyzed (S1 Table) were typeable by both MLTSA and MVLST. Because the clustering of the isolates was very similar using these two methods and because both the methods were based on sequences of short regions, these two methods were combined to obtain increased discriminatory power. As a result, while using only one of the methods generated 89 and 48 different sequence types with a Simpson’s index of diversity (DI) [25] of 0.976 and 0.933 for MLTSA and MVLST, respectively, combining these two methods generated 104 different sequence types with DI of 0.985 (Table 1). Depending on the source of origin, the 95 Japanese food and environmental isolates were discriminated into 56 subtypes, the 21 Japanese clinical isolates into 19 subtypes, and the 42 clinical isolates from other countries into 36 subtypes, indicating the good discriminatory power of this combined method for all isolate categories. This high DI value reflects the fact that approximately half of the sequence types obtained (50.6%) were represented by only one isolate.

**Table 1:** Allelic diversities of MLTSA with 3 regions and MVLST with 6 regions.

Method	Region	No. of alleles	Index of Diversity
MLTSA		89	0.976
	TR1	64	
	TR2	32	
	TR3	6	
MVLST		48	0.933
	*prfA*	8	
	*inlB*	13	
	*inlC*	17	
	*lisR*	5	
	*dal*	21	
	*clpP*	5	
MLSTA & MVLST		104	0.985

### Identification of food source of infection

Because of the long incubation period of *L*. *monocytogenes*, it is difficult to specify the food source of infection or link one case to another. Therefore, a significant number of listeriosis cases tend to be defined as sporadic cases as opposed to results of outbreaks. However, some retrospective genetic studies have confirmed past listeriosis outbreaks by identifying a number of genetically similar isolates from several clinical cases [26,27]. On the other hand, in the present study, there was great genetic diversity among Japanese clinical isolates; most of them (17/21) did not share MLTSA and MVLST profiles with any other Japanese clinical isolate, indicating that they are sporadic isolates.

**Fig 1:**
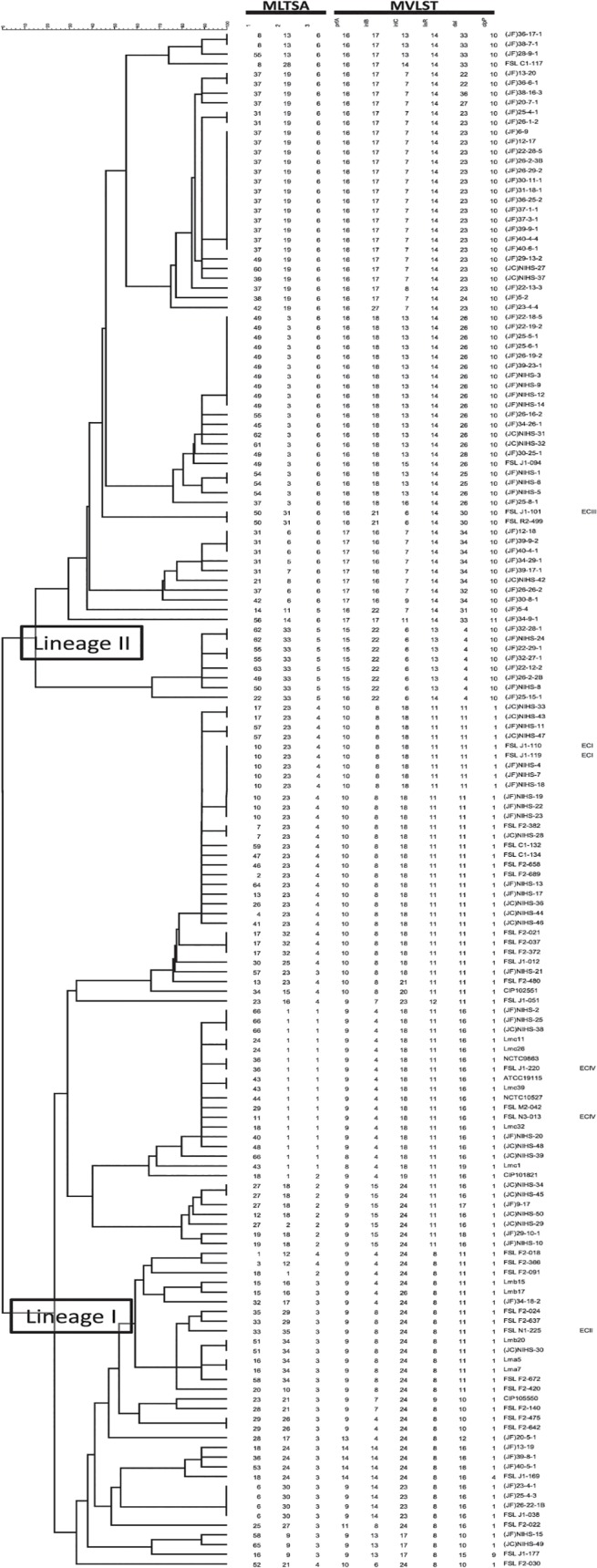
A dendrogram based on MLTSA and MVLST. The dendrogram is based on nucleotide sequences in 3 multilocus tandem-repeat sequence analysis (MLTSA) regions (regions 1, 2, and 3) and 6 multi-virulence-locus sequence typing (MVLST) regions. Epidemic clone groupings are shown to the right of the isolates. JF: Japanese food isolate, JC: Japanese clinical isolate.

Other than the clinical isolates, this study included a number of RTE seafood isolates collected in our laboratory [19,21]. Since the contamination rates of retailed RTE seafood, especially minced tuna and fish roe, are very high (5.7–12.1%) [21], these foods could be potential sources of foodborne listeriosis. Moreover, our previous *in vivo* and *in vitro* studies showed that Japanese RTE seafood isolates may have virulence potential that could pose a risk of causing foodborne listeriosis [21]. Therefore, the epidemiological relatedness of Japanese clinical isolates and RTE food isolates was then investigated. However, no Japanese clinical and RTE seafood isolates showed identical genotypes in this study (Fig 1), indicating that there was no epidemiological link between them.

However, although Japanese RTE seafood isolates were unlikely to be the source of past listeriosis cases, the possibility of listeriosis cases in the future cannot be denied. The Japanese RTE seafood isolates, 23-4-1, 25-4-3, and 26-22-1B, had the same genotype as FSL J1-038 (Fig 1), which is a human sporadic isolate from the United Sates. Sharing the same subtypes, as highlighted by this reliable and highly discriminatory subtyping method, can be indicative of the food isolates having an equal or similar level of virulence potential. Moreover, the genotypes of other RTE food isolates were not significantly different from genotypes of clinical isolates. This illustrates the necessity of vigilance for the future risk of listeriosis spread through contaminated RTE seafood in Japan.

### Prevalence of epidemic clone (EC)-related strains

Some geographically and temporally distinct human listeriosis outbreak isolates had been previously identified as “epidemic clones (EC)” because of their close genetic relatedness [6]. Chenal-Francisque et al. [28] conducted research on the worldwide distribution of *L*. *monocytogenes* by grouping isolates that shared at least six out of seven housekeeping gene sequences. According to their study, while ECI and ECIV were the cause of a number of foodborne outbreaks in many parts of the world and their closely related isolates were ubiquitous, ECII and ECIII consisted of isolates from the United States [29] and were rarely reported outside the United States, especially in Asian countries. Our present study included a number of EC isolates: ECI (FSL J1-110 and J1-119), ECII (FSL N1-225), ECIII (FSL J1-101), and ECIV (FSL J1-220 and FSL N3-013). Identical MVLST profiles showed that relatively large numbers of Japanese isolates were closely related to ECI and to ECIV (17 and 5 isolates for ECI and ECIV, respectively). Specifically, 6 Japanese food isolates shared their MLTSA and MVLST profiles with ECI isolates. On the other hand, ECII and ECIII-related isolates were rare among Japanese isolates (1 and 0 isolates for ECII and ECIII, respectively). Therefore, our study was in agreement with the study by Chenal-Francisque et al. [28] showing that ECII- and ECIII-related strains were not common in Japan.

### Suitability of MLTSA-MVLST combination method for typing *L*. *monocytogenes* isolates

In this study, MLTSA and MVLST were used for analyzing Japanese clinical and other *L*. *monocytogenes* isolates. MLTSA is a newly developed typing method based on three different tandem-repeat regions. While repeat numbers of short sequences in tandem-repeat regions are highly variable but stable among strains and have been used for typing many pathogens including *L*. *monocytogenes* [20,30–32], MLTSA detects even a single nucleotide variation in tandem-repeat regions, which are even more polymorphic. Moreover, this method could aid in accurate phylogenetic typing of *L*. *monocytogenes* isolates [17]. Chenal-Francisque et al. [33] evaluated 18 tandem repeat regions including TR1, TR2, and TR3 used in this study, and only TR2 was considered appropriate. The sequence variability of the flanking region of TR1 was an obstacle in their analysis, but we conducted a sequencing analysis rather than a fragment analysis for TR1, which is unaffected by the of sequence variability in the flanking region [32]. As for TR3, they had problem in amplifying this region, which may be because they used PCR conditions different from those optimized in our previous study [20]. MVLST, which was developed for typing *L*. *monocytogenes* strains on the basis of six virulence and virulence-associated genes, also has a high discriminatory power and suitability for phylogenetic analysis, and is therefore used widely [26,34,35]. Moreover, the high genomic stability of *L*. *monocytogenes* [36] contributes to the repeatability of these sequence-based methods. Pulsed-field gel electrophoresis (PFGE) is the gold standard for molecular subtyping of *L*. *monocytogenes* with the high discriminatory power. However, sequence-based methods such as MLTSA and MVLST are advantageous in that interpreting the data and sharing the results between laboratories is easier and more precise compared to fragment-based method, PFGE. Moreover, the discriminatory power of both these method are comparable to that of pulsed-field gel electrophoresis (PFGE) [17,18,20].

### Conclusions

This is, to the best of our knowledge, the first report comparing the genetic characteristics of Japanese clinical isolates with those of isolates of various sources. Japanese clinical listeriosis isolates were genetically variable, showing no specific trend or genetic similarity to isolates from other sources, which indicates that their occurrence is most probably from sporadic incidences. However, details of the isolates, such as the source, year, and place of isolation were not available, which would have been helpful in accurately determining whether they were from sporadic cases or were outbreak isolates that underwent subsequent genetic changes. Moreover, it is noteworthy that some of the Japanese clinical isolates (NIHS-28, NIHS-33, NIHS-36, NIHS-43, NIHS-44, NIHS-46, and NIHS-47) shared identical MVLST profiles with the ECI isolates. It is possible that Japanese clinical isolates with identical MVLST, but different MLTSA profiles, are from an outbreak, since tandem-repeat regions are highly changeable. In fact, two ECIV strains showed identical MVLST but different MLTSA profiles (Fig 1). Furthermore, it is also difficult to identify the infection source because of the long incubation period of *L*. *monocytogenes*. Even previous studies in which outbreaks were identified did not specify the source food, partially because of the time delay prior to research [26,27]. On the other hand, the infection source can be determined if a quick response is taken right after the listeriosis cases are detected [37,38]. In our previous study on the incidence and prevalence of *L*. *monocytogenes* in retailed RTE seafood in Japan, some RTE seafood isolates from different retailers of the same manufacturer had identical genotypes (unpublished data), indicating that contamination occurs during manufacturing, which is then spread by distribution of the contaminated foods and causes outbreaks over a wide geographic area. To prevent foodborne listeriosis cases in the future, the implementation of a mandatory reporting system and collection of isolates from clinical cases are necessary. Moreover, prompt investigation of a significant number of isolates using appropriate molecular typing should be adopted in Japan. It should also be noted that the combination of the two methods, MVLST and MLTSA, would be highly applicable for molecular typing, because of its high discriminatory power and accuracy in phylogenetic studies.

## Supporting Information

S1 Table The 158 *Listeria monocytogenes* isolates used in this study. (XLSX)

## References

[1] Makino SI, Kawamoto K, Takeshi K, Okada Y, Yamasaki M, Yamamoto S, et al An outbreak of food-borne listeriosis due to cheese in Japan, during 2001. Int J Food Microbiol. 2005; 104: 189–196. 15979181 10.1016/j.ijfoodmicro.2005.02.009

[2] Yamane K, Suzuki S, Shibayama K. Infect Agents Surveillance Rep (IASR). 2012; 33: 247–248. (In Japanese)

[3] Okutani A, Okada Y, Yamamoto S, Igimi S. Nationwide survey of human *Listeria monocytogenes* infection in Japan. Epidemiol Infect. 2004; 132:769–772. 15310181 10.1017/s0950268804001967 PMC2870160

[4] Farber JM, Peterkin PI. *Listeria monocytogenes*, a food-borne pathogen. Microbiol Mol Biol Rev. 1991; 55: 476–511. 10.1128/mr.55.3.476-511.1991 PMC372831 1943998

[5] Schuchat A, Swaminathan B, Broome CV. Epidemiology of human listeriosis. Clin Microbiol Rev. 1991; 4: 169–183. 1906370 10.1128/cmr.4.2.169 PMC358189

[6] Kathariou S. *Listeria monocytogenes* virulence and pathogenicity, a food safety perspective. J Food Prot. 2002; 65: 1811–1829. 12430709 10.4315/0362-028x-65.11.1811

[7] Liu D, Lawrence ML, Wiedmann M, Gorski L, Mandrell RE, Ainsworth AJ, et al *Listeria monocytogenes* subgroups IIIA, IIIB, and IIIC delineate genetically distinct populations with varied pathogenic potential. J Clin Microbiol. 2006; 44: 4229–4233. 17005751 10.1128/JCM.01032-06 PMC1698330

[8] Rasmussen OF, Skouboe P, Dons L, Rossen L, Olsen JE. *Listeria monocytogenes* exists in at least three evolutionary lines: evidence from flagellin, invasive associated protein and listeriolysin O genes. Microbiology. 1995; 141: 2053–2061. 7496516 10.1099/13500872-141-9-2053

[9] Roberts A, Nightingale K, Jeffers G, Fortes E, Kongo JM, Wiedmann M. Genetic and phenotypic characterization of *Listeria monocytogenes* lineage III. Microbiology. 2006; 152: 685–693. 16514149 10.1099/mic.0.28503-0

[10] Ward TJ, Ducey TF, Usgaard T, Dunn KA, Bielawski JP. Multilocus genotyping assays for single nucleotide polymorphism-based subtyping of *Listeria monocytogenes* isolates. Appl Environ Microbiol. 2008; 74: 7629–7642. 10.1128/AEM.01127-08 18931295 PMC2607178

[11] Wiedmann M, Bruce JL, Keating C, Johnson AE, McDonough PL, Batt CA. Ribotypes and virulence gene polymorphisms suggest three distinct *Listeria monocytogenes* lineages with differences in pathogenic potential. Infect Immun. 1997; 65: 2707–2716. 9199440 10.1128/iai.65.7.2707-2716.1997 PMC175382

[12] Gray MJ, Zadoks RN, Fortes ED, Dogan B, Cai S, Chen Y, et al Food and human isolates of *Listeria monocytogenes* form distinct but overlapping populations. Appl Environ Microbiol. 2004; 70: 5833–5841. 15466521 10.1128/AEM.70.10.5833-5841.2004 PMC522108

[13] Jeffers GT, Bruce JL, McDonough PL, Scarlet J, Boor KJ, Wiedmann M. Comparative genetic characterization of *Listeria monocytogenes* isolates from human and animal listeriosis cases. Microbiology. 2001; 147: 1095–1104. 11320113 10.1099/00221287-147-5-1095

[14] Norton DM, Scarlett JM, Horton K, Sue D, Thimothe J, Boor KJ, et al Characterization and pathogenic potential of *Listeria monocytogenes* isolates from the smoked fish industry. Appl Environ Microbiol. 2001; 67: 646–653. 11157227 10.1128/AEM.67.2.646-653.2001 PMC92631

[15] Franciosa G, Tartaro S, Wedell-Neergaard C, Aureli P. Characterization of *Listeria monocytogenes* strains involved in invasive and noninvasive listeriosis outbreaks by PCR-based fingerprinting techniques. Appl Environ Microbiol. 2001; 67: 1793–1799. 11282635 10.1128/AEM.67.4.1793-1799.2001 PMC92799

[16] Nightingale KK, Windham K, Wiedmann M. Evolution and molecular phylogeny of *Listeria monocytogenes* isolated from human and animal listeriosis cases and foods. J Bacteriol. 2005; 187: 5537–5551. 16077098 10.1128/JB.187.16.5537-5551.2005 PMC1196091

[17] Miya S, Takahashi H, Kamimura C, Nakagawa M, Kuda T, Kimura B. Highly discriminatory typing method for *Listeria monocytogenes* using polymorphic tandem repeat regions. J Microbiol Met. 2012; 90: 285–291. 10.1016/j.mimet.2012.05.023 22677602

[18] Zhang W, Jayarao BM, Knabel SJ. Multi-virulence-locus sequence typing of *Listeria monocytogenes* . Appl Environ Microbiol. 2004; 70: 913–920. 14766571 10.1128/AEM.70.2.913-920.2004 PMC348834

[19] Handa S, Kimura B, Takahashi H, Koda T, Hisa K, Fujii T. Incidence of *Listeria monocytogenes* in raw seafood products in Japanese retail stores. J Food Prot. 2005; 68: 411–415. 15726989 10.4315/0362-028x-68.2.411

[20] Miya S, Kimura B, Sato M, Takahashi H, Ishikawa T, Suda T, et al Development of a multilocus variable-number of tandem repeat typing method for *Listeria monocytogenes* serotype 4b strains. Int J Food Microbiol. 2008; 124: 239–249. 10.1016/j.ijfoodmicro.2008.03.023 18457891

[21] Miya S, Takahashi H, Ishikawa T, Fujii T, Kimura B. Risk of *Listeria monocytogenes* contamination of raw ready-to-eat seafood products available at retail outlets in Japan. Appl Environ Microbiol. 2010; 76: 3383–3386. 10.1128/AEM.01456-09 20348310 PMC2869148

[22] Takahashi H, Handa-Miya S, Kimura B, Sato M, Yokoi A, Goto S, et al Development of multilocus single strand conformation polymorphism (MLSSCP) analysis of virulence genes of *Listeria monocytogenes* and comparison with existing DNA typing methods. Int J Food Microbiol. 2007; 118: 274–284. 17822795 10.1016/j.ijfoodmicro.2007.07.047

[23] Ward TJ, Gorski L, Borucki MK, Mandrell RE, Hutchins J, Pupedis K. Intraspecific phylogeny and lineage group identification based on the *prfA* virulence gene cluster of *Listeria monocytogenes* . J Bacteriol. 2004; 186: 4994–5002. 15262937 10.1128/JB.186.15.4994-5002.2004 PMC451661

[24] Maiden MCJ, Bygraves JA, Feil E, Morelli G, Russell JE, Urwin R, et al Multilocus sequence typing: a portable approach to the identification of clones within populations of pathogenic microorganisms. Proc Natl Acad Sci USA. 1998; 95: 3140–3145. 9501229 10.1073/pnas.95.6.3140 PMC19708

[25] Hunter PR, Gaston MA. Numerical index of the discriminatory ability of typing systems: an application of Simpson’s index of diversity. J Clin Microbiol. 1988; 26: 2465–2466. 3069867 10.1128/jcm.26.11.2465-2466.1988 PMC266921

[26] Knabel SJ, Reimer A, Verghese B, Lok M, Ziegler J, Farber J, et al Sequence typing confirms that a predominant *Listeria monocytogenes* clone caused human listeriosis cases and outbreaks in Canada from 1988 to 2010. J Clin Microbiol. 2012; 50: 1748–1751. 10.1128/JCM.06185-11 22337989 PMC3347097

[27] Yde M, Botteldoorn N, Bertrand S, Collard JM, Dierick K. Microbiological and molecular investigation of an increase of human listeriosis in Belgium, 2006–2007. Euro Surveill. 2010; 15: 19482 20158978

[28] Chenal-Francisque V, Lopez J, Cantinelli T, Caro V, Tran C, Leclercq A, et al Worldwide distribution of major clones of *Listeria monocytogenes* . Emerg Infect Dis. 2011; 17: 1110–1112. 10.3201/eid/1706.101778 21749783 PMC3358213

[29] Kathariou S, Graves L, Buchrieser C, Glaser P, Siletzky RM, Swaminathan B. Involvement of closely related strains of a new clonal group of *Listeria monocytogenes* in the 1998–99 and 2002 multistate outbreaks of foodborne listeriosis in the United States. Foodborne Pathog Dis. 2006; 3: 292–302. 16972778 10.1089/fpd.2006.3.292

[30] Lindstedt BA, Tham W, Danielsson-Tham ML, Vardund T, Helmersson S, Kapperud G. Multiple-locus variable-number tandem-repeats analysis of *Listeria monocytogenes* using multicolour capillary electrophoresis and comparison with pulsed-field gel electrophoresis typing. J Microbiol Methods. 2008; 72: 141–148. 18096258 10.1016/j.mimet.2007.11.012

[31] Murphy M., Corcoran D, Buckley JF, O’Mahony M., Whyte P, Fanning S. Development and application of multiple-locus variable number of tandem repeat analysis (MLVA) to subtype a collection of *Listeria monocytogenes* . Int J Food Microbiol. 2007; 115: 187–194. 17174430 10.1016/j.ijfoodmicro.2006.10.022

[32] Volpe Sperry KE, Kathariou S, Edwards JS, Wolf LA. Multiple-locus variable-number tandem-repeat analysis as a tool for subtyping *Listeria monocytogenes* strains. J Clin Microbiol. 2008; 46: 1435–1450. 10.1128/JCM.02207-07 18256218 PMC2292909

[33] Chenal-Francisque V, Diancourt L, Cantinelli T, Passet V, Tran-Hykes C, Bracq-Dieye H, et al Optimized multilocus variable- number tandem-repeat analysis assay and its complementarity with pulsed-field gel electrophoresis and multilocus sequence typing for *Listeria monocytogenes* clone identification and surveillance. J. Clin. Microbiol. 2013; 51: 1868–1880. 10.1128/JCM.00606-13 23576539 PMC3716049

[34] Chen Y, Zhang W, Knabel SJ. Multi-virulence-locus sequence typing clarifies epidemiology of recent listeriosis outbreaks in the United States. J Clin Microbiol. 2005; 43: 5291–5294. 16208000 10.1128/JCM.43.10.5291-5294.2005 PMC1248515

[35] Handa-Miya S, Kimura B, Takahashi H, Sato M, Ishikawa T, Igarashi K, et al Nonsense-mutated *inlA* and *prfA* not widely distributed in *Listeria monocytogenes* isolates from ready-to-eat seafood products in Japan. Int J Food Microbiol. 2007; 117: 312–318. 17566579 10.1016/j.ijfoodmicro.2007.05.003

[36] Nelson KE, Fouts DE, Mongodin EF, Ravel J, DeBoy RT, Kolonay JF, et al Whole genome comparisons of serotype 4b and 1/2a strains of the food-borne pathogen *Listeria monocytogenes* reveal new insights into the core genome components of this species. Nucleic Acids Res. 2004; 32:2386–2395. 15115801 10.1093/nar/gkh562 PMC419451

[37] Gottlieb SL, Newbern EC, Griffin PM, Graves LM, Hoekstra RM, Baker NL, et al Multistate outbreak of listeriosis linked to turkey deli meat and subsequent changes in US regulatory policy. Clin Infect Dis. 2006; 42: 29–36. 16323088 10.1086/498113

[38] Olsen SJ, Patrick M, Hunter SB, Reddy V, Kornstein L, MacKenzie WR, et al Multistate outbreak of *Listeria monocytogenes* infection linked to delicatessen Turkey Meat. Clin Infect Dis. 2005; 40: 962–967. 15824987 10.1086/428575

